# Serum sex hormones correlate with pathological features of papillary thyroid cancer

**DOI:** 10.1007/s12020-023-03554-w

**Published:** 2023-10-10

**Authors:** Fa-Zhan Xu, Lu-Lu Zheng, Ke-Hao Chen, Ru Wang, Dan-Dan Yi, Chao-Yu Jiang, Zhi-Jian Liu, Xian-Biao SHI, Jian-Feng Sang

**Affiliations:** 1 Nanjing Drum Tower Hospital Clinical College of Nanjing Medical University, Nanjing, China; 2 Division of Thyroid Surgery, Department of General Surgery, Nanjing Drum Tower Hospital, the Affiliated Hospital of Medical School, Nanjing University, Nanjing, 210008 China

**Keywords:** Papillary Thyroid Cancer, Sex Hormone, Testosterone, Estradiol

## Abstract

**Purpose:**

Sex hormones are thought to be responsible for the unique gender differences in papillary thyroid cancer(PTC). Most previous studies on these have focused on the expression of estrogen receptors, or have been limited to animal studies. The aim of our study was to explore the relationship between serum sex hormones and the pathological features of PTC in the clinical setting, as further evidence of the role of sex hormones in PTC.

**Methods:**

Retrospective data analysis of patients who underwent thyroid surgery at the Department of Thyroid Surgery, Nanjing Drum Tower Hospital from January 2022 to September 2022 Correlation between serum sex hormone and pathological features was analyzed in male patients and in menopausal female patients. Serum sex hormones include luteinizing hormone(LH), follicle stimulating hormone(FSH), estradiol(E_2_), total testosterone(TT), progesterone(P), and prolactin(PRL). Tumor pathological characteristics include the number and size of tumor, presence of extrathyroidal extension(ETE), presence of lymph node metastasis(LNM).

**Results:**

Preoperative serum E2 in male patients was positively correlated with tumor size in PTC, LH was negatively correlated with LNM, while TT and P were negatively correlated with ETE. Similar findings were not observed in menopausal female patients.

**Conclusion:**

We observed that serum sex hormones correlate with the pathological features of PTC in male patients, for the first time in a clinical study. High serum estrogens may be a risk factor for PTC, while androgens are the opposite. This somewhat corroborates previous research and provides new variables for future PTC prediction models.

## Introduction

Thyroid carcinoma (TC) is the most common malignancy of the endocrine system, with papillary thyroid carcinoma (PTC) as the main pathological strain. It is the seventh most common malignancy in women, with an incidence rate of 2.8 times that of men and an average age at disease less than that of men [1], and male sex is a risk factor for poor prognosis in TC [2]. China has the highest number of TC cases and deaths in the world, but its unique gender differences have not been articulated [3].

Sex hormones are the possible explanation. Previous studies have identified estrogen receptors, androgen receptors, prolactin receptors, and progesterone receptors expressed in PTC lesions [4, 5]. It is now believed that estrogens can regulate the development of PTC through genomic or non-genomic effect [6], while androgens have been suggested to inhibit the progression of PTC in recent studies [7, 8]. These findings suggest that sex hormones may provide new clinical strategies for papillary thyroid cancer, such as prediction of diagnosis, endocrine therapy, and assessment of prognostic risk, as in breast cancer and prostate cancer. However, the results of these studies are mainly based on in vitro cellular experiments or animal experiments and fail to have sufficient evidence for clinical studies. This may be due to the large fluctuation of sex hormones in non-menopausal female patients, resulting in few clinical studies directly focusing on the association of serum sex hormones with PTC.

In this study, we retrospectively analyzed for the first time the correlation between serum sex hormones and pathological characteristics in two subgroups of clinically male PTC patients and menopausal female PTC patients to further explore the role of sex hormones in PTC and to provide more ideas for the treatment of PTC.

## Materials and methods

### Data sources

We retrospectively analyzed data from patients with pathologically confirmed PTC who underwent thyroid surgery from January 2022 to September 2022 at the Division of Thyroid Surgery, Department of General Surgery, Nanjing Drum Tower Hospital. Considering that patients with a history of previous thyroid surgery have altered thyroid function, patients with incomplete data and a history of previous thyroid surgery were excluded. 446 patients were eligible and included in this study. All patients met the inclusion criteria: (1) First thyroid surgery. (2) Postoperative pathological findings confirmed PTC. (3) Complete clinical data. As this was a retrospective study, all data were obtained from previous medical records and used only by the investigators, the Ethics Committee of Nanjing Drum Tower Hospital approved the exemption of informed consent for this study (decision no: 2023-345-01).

### Data collection

All patients were tested for serum thyroid function and sex hormones preoperatively. In this study, we collected mainly gender, age, body mass index, thyroid stimulating hormone (TSH), thyroglobulin (Tg), sex hormones, and postoperative pathology of the patients. Serum sex hormone levels were measured by chemiluminescence (CL) measurements and thyroid function levels were measured by electrochemiluminescence (ECL) measurements at the Department of Nuclear Medicine, Nanjing Drum Tower Hospital, Nanjing, China. Serum sex hormones included estradiol (E_2_), total testosterone (TT), luteinizing hormone (LH), follicle stimulating hormone (FSH), and progesterone (P). Postoperative pathology included the following clinicopathological characteristics: number and size of tumors, presence of extrathyroidal extension (ETE), and presence of lymph node metastases (LNM). Multifocality was defined as two or more tumors in the thyroid, and tumor size was defined as the maximum diameter of the largest tumor. ETE was defined as microscopic invasion of the surrounding fibrofatty connective tissue, muscle tissue or visual finding of lesions into the strap muscles, larynx, trachea, esophagus, recurrent laryngeal nerve, prevertebral fascia, encircling carotid artery or mediastinal vessels.

### Statistical analysis

Data were analyzed with SPSS 26.0 (SPSS Inc., Chicago, USA). Categorical variables in the univariate analysis were tested by chi-square test, and measures that met normality were tested by t-test, and vice versa by rank sum test. For correlation analysis of tumor size, Spearman correlation analysis was used, and variables with *P* < 0.1 in the correlation analysis were subjected to one-way linear regression analysis, and finally variables with *P* < 0.1 in the one-way linear regression analysis were included in the stepwise method for multiple linear regression analysis. Correlation analyses of pathological characteristics were performed using binary logistic regression, incorporating variables with *P* < 0.1 in the one-way analysis, and stepwise regression analysis using Forward: Conditional. Significant variables were analyzed using receiver operating characteristic curve (ROC). *P* < 0.05 was considered a statistically significant difference.

## Results

### General characteristics of PTC patients

Of the 446 PTC patients, 318 were female and 128 were male (female-to-male ratio of 5:2), and the mean age was 42.67 ± 11.70 years old (age ranging from 16 to 75 years old), male patients had a slightly higher BMI than female patients (*P* < 0.001), while female patients had a higher TSH (*P* = 0.009) than male patients and were also more likely to have lymph node metastasis (*P* < 0.001) (Table 1). When female patients were grouped according to menopausal status, non-menopausal female patients were more likely to have lymph node metastases than menopausal female patients (*P* = 0.034). Gender and menopause did not affect tumor size or ETE.

**Table 1:** Characteristics of patients with PTC in different gender

Variables	Total *N* = 446	Gender			Female		
		Male *N* = 128	Female *N* = 318	*P* value	Non-menopausal *N* = 218	Menopausal *N* = 100	*P* value
Age, mean ± SD, years	42.67 ± 11.70	41.42 ± 10.98	43.17 ± 11.94	0.155	36.76 ± 7.75	57.13 ± 6.19	<0.001
BMI, mean ± SD	24.29 ± 3.70	25.56 ± 3.60	23.78 ± 3.61	<0.001	23.39 ± 3.68	24.63 ± 3.30	0.004
TSH, mean ± SD, mIU/L	2.37 ± 1.67	2.04 ± 1.93	2.50 ± 1.54	0.009	2.61 ± 1.49	2.25 ± 1.63	0.066
Tg, mean ± SD, ng/mL	31.03 ± 48.49	32.21 ± 52.78	30.55 ± 46.64	0.744	28.32 ± 41.53	35.41 ± 55.90	0.209
LH, mean ± SD, mIU/mL	11.69 ± 14.70	3.57 ± 1.93	14.96 ± 16.26	<0.001	9.44 ± 14.88	27.01 ± 12.09	<0.001
E_2_, mean ± SD, pmol/L	256.30 ± 237.72	147.72 ± 45.59	300.00 ± 267.89	<0.001	388.88 ± 274.00	106.26 ± 98.95	<0.001
FSH, mean ± SD, mIU/mL	20.67 ± 28.58	6.02 ± 4.60	26.57 ± 31.87	<0.001	10.21 ± 18.18	62.22 ± 25.60	<0.001
PRL, mean ± SD, ug/L	16.40 ± 12.27	12.38 ± 5.27	18.01 ± 13.81	<0.001	20.65 ± 12.91	12.27 ± 13.99	<0.001
P, mean ± SD nmol/L	6.12 ± 10.73	2.06 ± 0.68	7.75 ± 12.33	<0.001	10.56 ± 14.01	1.61 ± 0.81	<0.001
TT, mean ± SD, nmol/L	4.16 ± 5.90	12.32 ± 5.16	0.85 ± 0.30	<0.001	0.91 ± 0.30	0.71 ± 0.23	<0.001
Tumer size, mean ± SD, cm	1.06 ± 0.73	1.17 ± 0.85	1.01 ± 0.66	0.064	0.99 ± 0.65	1.06 ± 0.70	0.346
Multifocality, n(%)	186 (41.61)	48 (37.5)	138 (43.4)	0.253	90 (41.28)	48 (48.00)	0.262
LNM, n(%)	265 (59.28)	94 (73.44)	171 (53.77)	<0.001	126 (57.8)	45 (45.00)	0.034
ETE, n(%)	297 (66.44)	85 (66.41)	(212) 66.67	0.958	144 (66.06)	68 (68.00)	0.733

**Fig. 1:**
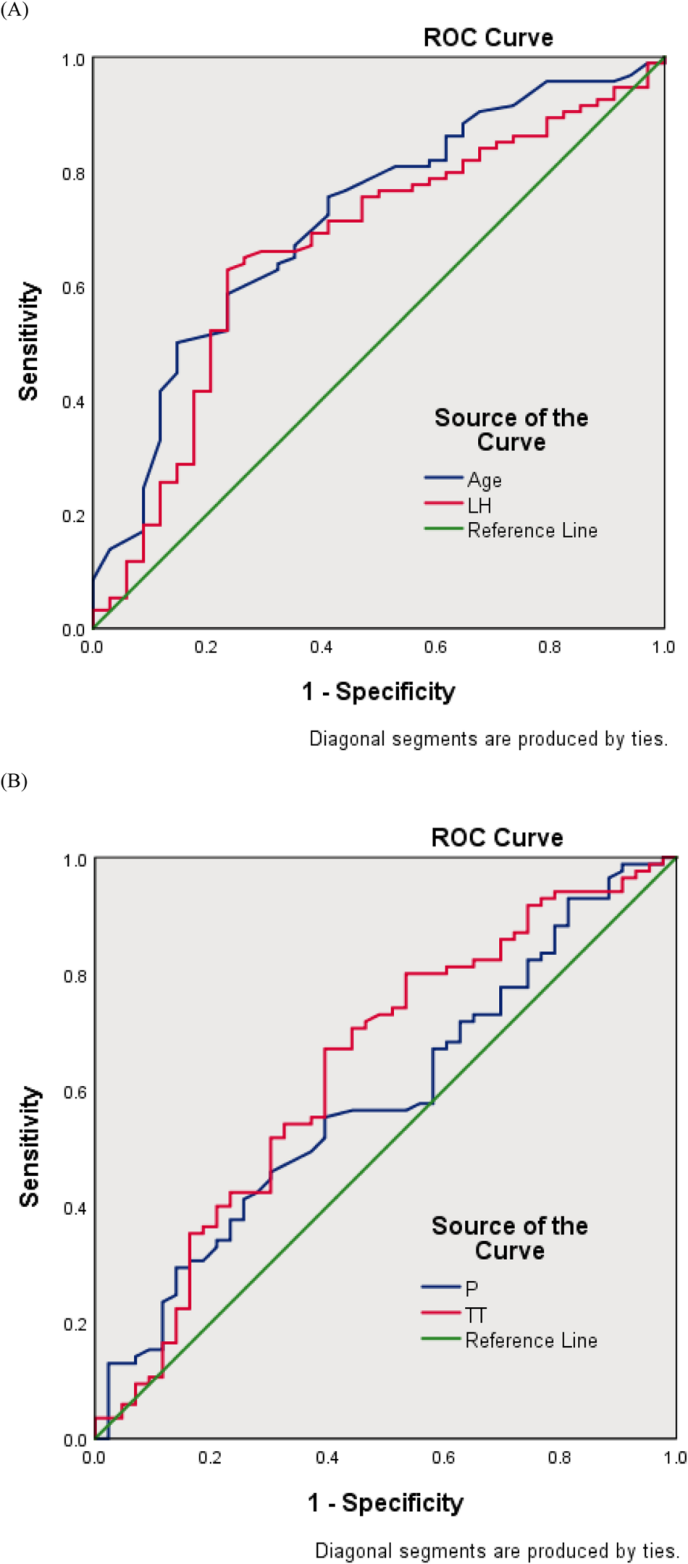
ROC curves. **A** ROC curve for the correlation of CLNM with age and LH. The ROC results showed that the AUC for Age was 0.714, 95% CI was 0.615–0.814, *P* < 0.001, and the AUC for LH was 0.666, 95% CI was 0.599–0.739, *P* = 0.004. **B** ROC curve for the correlation of ETE with TT and P. The ROC results showed that the AUC for P was 0.584, 95% CI was 0.480–0.687, *P* = 0.123, the AUC for TT was 0.637, 95% CI was 0.532–0.742, *P* = 0.012

### Correlation analysis of serum sex hormones and pathological features of PTC

Considering that the sex hormone s of non-menopausal female patients fluctuate with the menstrual cycle, while those of male patients and menopausal female patients are more stable, we mainly analyzed two subgroups, male patients and menopausal female patients, to investigate the relationship between serum sex hormones and tumor pathological characteristics. Considering that previous studies have reported tumor size as an independent risk factor for ETE and LNM [9, 10], in order to analyze the independent role of sex hormones, we included tumor size in the model for multifactorial correlation analysis.

#### Correlation between sex hormones and tumor size in male PTC patients

The results of multifactorial analysis showed that preoperative serum E2 was positively correlated with tumor size in male patients (Table 2). In addition to this, we also observed that serum Tg was also positively correlated with tumor size in male patients, while age was negatively correlated.

**Table 2:** Correlation analysis of age, BMI, TSH, Tg, serum sex hormone levels and tumor size in male patients

	Correlation analysis	Single-factor linear regression analysis		Multiple linear regression analysis	
	*P* value	B[95% CI]	*P* value	B[95% CI]	*P* value
Age	<0.001	−0.023 [−0.036–0.010]	0.001	−0.012 [−0.024–0.000]	0.044
BMI	0.886	–	–	–	
TSH	0.603	–	–	–	
Tg	<0.001	0.008 [0.006–0.011]	<0.001	0.008 [0.005–0.010]	<0.001
LH	0.760	–	–	–	
E_2_	0.067	0.003 [0.000–0.007]	0.049	0.003 [0.000–0.006]	0.037
FSH	0.671	–	–	–	
PRL	0.838	–	–	–	
P	0.978	–	–	–	
TT	0.036	−0.018 [−0.047–0.011]	0.217	–	

#### Correlation between serum sex hormones and pathological characteristics of tumors in male patients

We analyzed the correlation between age, BMI, preoperative TSH, Tg, and serum sex hormones and tumor pathological characteristics in male patients (Table 3). The results of multifactorial logistic regression analysis showed that TT was negatively correlated with ETE of the tumor, as was P, while LH was positively correlated with LNM. In addition we observed that BMI was positively correlated with multifocality in male patients, while age was negatively correlated with LNM.

**Table 3:** Correlation analysis of age, BMI, TSH, Tg, serum sex hormone levels and pathological characteristics of tumors in male patients

	Multifocality			LNM			ETE		
	Single Factor	Multi-Factor		Single Factor	Multi-Factor		Single Factor	Multi-Factor	
	*P* value	OR[95% CI]	*P* value	*P* value	OR[95% CI]	*P* value	*P* value	OR[95% CI]	*P* value
Age	0.547			<0.001	0.945 [0.907–0.985]	0.008	0.890		
BMI	0.019	1.138 [1.020–1.269]	0.021	0.330			0.416		
TSH	0.664			0.886			0.146		
Tg	0.133			0.095			0.185		
Tumor size	0.039	1.598 [1.020–2.503]	0.041	0.011	2.989 [1.136–7.866]	0.027	0.012	2.434 [1.154–5.136]	0.019
LH	0.676			0.032	0.784 [0.630–0.977]	0.030	0.392		
E_2_	0.028			0.330			0.450		
FSH	0.384			0.050			0.536		
PRL	0.158			0.196			0.545		
P	0.442			0.395			0.081	0.507 [0.276–0.934]	0.029
TT	0.792			0.121			0.017	0.901 [0.831–0.978]	0.013

#### Correlation between serum sex hormone s and tumor size in menopausal female patients

We performed the same analysis as before on data from menopausal female patients, but failed to observe an association between serum sex hormones and tumor size (Table 4). However, in menopausal female patients we also observed a positive association between Tg and tumor size.

**Table 4:** Correlation analysis of age, BMI, TSH, Tg, serum sex hormone levels and tumor size in menopausal female patients

	Correlation analysis	Single-factor linear regression analysis		Multiple linear regression analysis	
	*P* value	B[95% CI]	*P* value	B[95% CI]	*P* value
Age	0.033	0.012 [−0.010–0.035]	0.272		
BMI	0.062	0.005–0.087	0.030		
TSH	0.863				
Tg	0.003	0.003 [0.001–0.006]	0.009	0.003 [0.001–0.006]	0.009
LH	0.047	−0.009 [−0.021–0.002]	0.111		
E_2_	0.435				
FSH	0.044	−0.005 [−0.010–0.000]	0.062		
PRL	0.178				
P	0.201				
TT	0.499				

#### Correlation between serum sex hormones and pathological characteristics of tumors in menopausal female patients

We analyzed data from menopausal female patients in the same way and did not observe an independent correlation between serum sex hormones and the pathological characteristics of the tumor (Table 5).

**Table 5:** Correlation analysis of age, BMI, TSH, Tg, serum sex hormone levels and pathological characteristics of tumors in menopausal female patients

	Multifocality			LNM			ETE		
	Single Factor	Multi-Factor		Single Factor	Multi-Factor		Single Factor	Multi-Factor	
	*P* value	OR[95% CI]	*P* value	*P* value	OR[95% CI]	*P* value	P value	OR[95% CI]	*P* value
Age	0.942			0.944			0.214		
BMI	0.290			0.565			0.293		
TSH	0.300			0.732			0.677		
Tg	0.520			0.795			0.027	1.026 [1.003–1.050]	0.027
Tumor size	0.139			<0.001	5.001 [2.023–12.364]	<0.001	0.183		
LH	0.365			0.299			0.261		
E_2_	0.769			0.630			0.146		
FSH	0.512			0.897			0.848		
PRL	0.355			0.484			0.307		
P	0.556			0.269			0.831		
TT	0.831			0.640			0.929		

#### ROC curve analysis

The previous analysis showed that in male patients, LH and age were negatively correlated with LNM, while TT and P were negatively correlated with ETE, and we further analyzed the correlations using ROC curves (Fig. 1). The results showed that in male patients, age had a diagnostic value for predicting LNM (AUC = 0.714, *P* < 0.001) and LH had some diagnostic value for predicting LNM (AUC = 0.666, *P* = 0.004). TT also had some diagnostic value for predicting ETE (AUC = 0.637, *P* = 0.012).

## Discussion

Given that serum sex hormones in non-menopausal women fluctuate with the menstrual cycle and that previous studies have focused on sex hormone receptors [11–16], we explored for the first time the relationship between all serum sex hormones and tumor pathology male patients and in menopausal female patients clinically. The results suggest sex hormones did independently influence tumor development in male patients, but failed to observe consistency in menopausal female patients. This may be due to the fact that sex hormones in the menopausal state are not representative of levels throughout the life cycle. Considering that PTC occurs in non-menopausal women, the question of how to analyze the correlation between sex hormones and PTC pathology in non-menopausal women is currently unresolved.

Previous studies have shown that estrogen promotes the development of PTC via the estrogen receptor (ER) [16]. ERα dose-dependently stimulates the survival and growth of PTC tumor cells. E_2_ has been shown to significantly elevate ERα expression in TC cell lines and promote TC proliferation via the RAS/RAF/MAPK/ERK pathway [6]. This is consistent with the results we observed. In our study, E_2_ levels in male patients were positively correlated with tumor size. In vitro and animal experiments have shown that estrogen promotes EMT and migration of PTC cells [12], however, in our results failed to observe a clinical correlation of serum E_2_ with multifocality, ETE and LNM.

Only a few studies have focused on the role of androgens and the androgen receptor (AR) in TC, and some of the findings are contradictory. Androgens act through the expression of AR in TC [13]. Early studies suggested that testosterone induces proliferation in PTC and follicular thyroid cancer (FTC) cell lines in in vitro experiments [17]. Neutered male mice have smaller FTCs than non-neutered males [18]. Recent studies have shown that androgen-AR overexpression inhibits the migratory activity and invasive behavior of PTC cells [13], as well as the expression of programmed death receptor ligand 1 (PD-L1) in in vitro studies [8]. Activation of AR induces senescence of PTC cells [7]. Dihydrotestosterone (DHT) induces G1 arrest in androgen-responsive thyroid cancer cells [19]. Our data suggest that higher androgens have an inhibitory effect on ETE in male patients clinically. This further corroborates the antitumor effect of androgens in PTC.

However, our study also revealed some unique findings. Our study showed that serum LH was negatively correlated with LNM in male patients. Previous studies have shown that LH receptors are expressed in PTC tissues [20]. Since the structure of LH is similar to TSH, LH has a weak thyroid stimulating effects [21]. β subunits of LH bind to TSH receptors and activate thyroid function in vitro [22]. This seems to suggest that LH is similar to TSH and can promote the development of PTC. It cannot explain our results which needs to be further explored. In addition, we observed a negative correlation between serum P and ETE in male patients. Several studies have reported that progesterone receptor (PR) expression was observed in paraffin-embedded PTC specimens [15], that PR expression was higher in PTC cells compared with para-cancerous tissue, and that higher rates of local metastasis were observed in patients with PTC expressing PR [23]. Overall, very few studies have focused directly on the relationship between serum LH, P, and PTC, and the observations in our study may be one-sided, and the mechanisms involved have failed to be reasonably elucidated. We plan to validate the findings of this study in a larger population.

The correlation between sex hormones and tumor pathological features was further analyzed using ROC curves, which suggested that LH and age have some diagnostic value for predicting LNM and TT for predicting ETE. This may provide new variables for future diagnostic and prognostic models of PTC.

Our study has some limitations. First, our data came from one center and the sample size was small. Second, this study only analyzed the clinical data of male patients and menopausal female patients, and we have not found a way to analyze non-menopausal female patients. Third, the information collected in this study was insufficient and we did not collect information on the number of metastatic lymph nodes or the extent of ETE. In addition, this study focused only on serum sex hormone levels, which would further refine our conclusions if combined with sex hormone receptor expression in PTC lesions.

In summary, we have observed in our clinical studies that serum sex hormones are involved in regulating the development of PTC. Especially in male patients, androgens play an anti-invasive role in PTC progression, while estrogens contribute to the development of PTC. This may provide a new idea for the treatment of PTC. However, we also observed that men are more likely to develop lymph node metastasis, suggesting that sex differences in PTC are not only determined by sex hormones. There are some studies suggesting that these sex differences may also be genetically and chromosomally related [24, 25], although no uniform results have been developed yet, which requires further research.

## Conclusion

In conclusion, we observed that serum sex hormones correlate with the pathological features of PTC, for the first time in a clinical study. Androgens were negatively correlated with ETE of PTC, while estrogens were positively correlated with tumor size, especially in male patients, which corroborates previous in vitro and animal studies. High serum estrogens may be a risk factor for PTC, while androgens are the opposite. This provides new variables for future diagnostic and prognostic models and a new idea for the treatment of PTC.
